# Leveraging Engineering of Indocyanine Green-Encapsulated Polymeric Nanocomposites for Biomedical Applications

**DOI:** 10.3390/nano8060360

**Published:** 2018-05-24

**Authors:** Ya-Hui Han, Ranjith Kumar Kankala, Shi-Bin Wang, Ai-Zheng Chen

**Affiliations:** 1 Institute of Biomaterials and Tissue Engineering, Huaqiao University, Xiamen 361021, China; ka_han@outlook.com (Y.-H.H.); sbwang@hqu.edu.cn (S.-B.W.); 2 College of Chemical Engineering, Huaqiao University, Xiamen 361021, China; 3 Fujian Provincial Key Laboratory of Biochemical Technology, Xiamen 361021, China

**Keywords:** indocyanine green, polymeric carriers, drug delivery, imaging, photodynamic therapy

## Abstract

In recent times, photo-induced therapeutics have attracted enormous interest from researchers due to such attractive properties as preferential localization, excellent tissue penetration, high therapeutic efficacy, and minimal invasiveness, among others. Numerous photosensitizers have been considered in combination with light to realize significant progress in therapeutics. Along this line, indocyanine green (ICG), a Food and Drug Administration (FDA)-approved near-infrared (NIR, >750 nm) fluorescent dye, has been utilized in various biomedical applications such as drug delivery, imaging, and diagnosis, due to its attractive physicochemical properties, high sensitivity, and better imaging view field. However, ICG still suffers from certain limitations for its utilization as a molecular imaging probe in vivo, such as concentration-dependent aggregation, poor in vitro aqueous stability and photodegradation due to various physicochemical attributes. To overcome these limitations, much research has been dedicated to engineering numerous multifunctional polymeric composites for potential biomedical applications. In this review, we aim to discuss ICG-encapsulated polymeric nanoconstructs, which are of particular interest in various biomedical applications. First, we emphasize some attractive properties of ICG (including physicochemical characteristics, optical properties, metabolic features, and other aspects) and some of its current limitations. Next, we aim to provide a comprehensive overview highlighting recent reports on various polymeric nanoparticles that carry ICG for light-induced therapeutics with a set of examples. Finally, we summarize with perspectives highlighting the significant outcome, and current challenges of these nanocomposites.

## 1. Introduction

**Figure 1:**
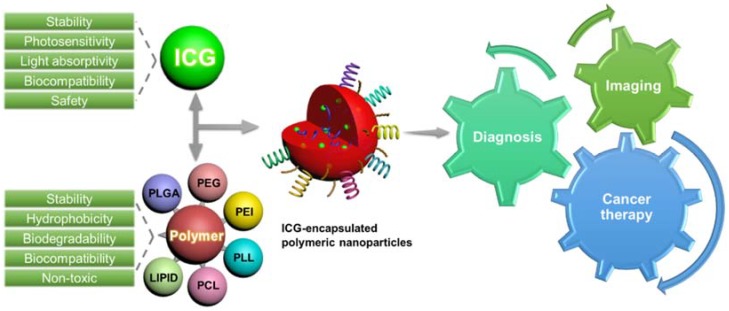
Schematic illustration highlighting the importance of various indocyanine green (ICG)-encapsulated polymeric nanoconstructs that have been utilized for various potential applications such as drug delivery, imaging, and diagnosis.

Since antiquity, the light from various sources (natural sun light, flare, and lightning, and artificial-laser) has been applied in a wide range of fields, including clean energy, electronics, optical fiber communications, and health care, among others [1]. Along this line, light-based therapeutics have gathered enormous interest from researchers in the past two decades for various biomedical applications such as diagnosis, drug delivery, and imaging, among others. These treatment strategies, particularly photothermal therapy (PTT) and photodynamic therapy (PDT) [1], have emerged as promising alternatives to the traditional chemotherapeutic approaches and surgery practices, due to beneficial properties such as minimal invasiveness [2,3,4], high efficacy [3,4], preferential localization [5,6], excellent tissue penetration [7,8,9,10], and improved patient compliance [2,4,5,11,12]. Oftentimes these strategies are controlled by using specific sensitizers that are preferentially located at the desired site and subsequently activated in the presence of light to perform their therapeutic duties. Conceptually, these photosensitizers are activated by light at its specific wavelength window to exhibit the desired therapeutic effect through various mechanisms [13]. However, the applicability of these approaches is limited in some instances as they predominantly depend on the location of treatment and poor penetration [14,15]. Among various photosensitizers, indocyanine green (ICG), a Food and Drug Administration (FDA)-approved near-infrared (NIR, >750 nm) fluorescent dye, has received considerable attention in various biomedical applications such as drug delivery, imaging, and diagnosis, due to its attractive physicochemical properties [16], extremely high sensitivity, and better imaging view field (Figure 1) [17]. The physicochemical properties of ICG, such as solubility, structural elucidations, absorption window, and metabolic features, explicitly provide information on its behavior and performance in vivo are comprehensively discussed hereunder.

ICG is an amphiphilic NIR fluorescent tricarbocyanine dye (Chemical formula—C_43_H_47_N_2_NaO_6_S_2_, Molecular Weight = 774.97 (*m*/*z* = 774.28)) that possesses two polycyclic moieties, which contribute to its high lipophilicity, and a sulfate group connected to each polycyclic ring for its hydrophilic properties [18,19]. The amphiphilic property of ICG facilitates the enhancement of its solubility in physiological fluids and its bioavailability [20,21]. It should be noted that thermal or light-induced degradation may cause its instability in an aqueous environment [22]. In addition, this dye possesses other attractive features, such as safety [23,24,25,26], good signal-to-noise ratio, and is inexpensive [26,27,28]. Moreover, ICG also exhibits promising optical properties, which vary with the components in the surrounding environment. The enormous light absorption capacity of ICG and its absorption spectrum utterly depend on various crucial factors such as concentration, solvent, excitation light spectra, and the filter used for detection [18,22,29,30]. In general, ICG efficiently absorbs the infrared (IR) light at a specific wavelength window ranging from 740 to 800 nm [17]. In aqueous solutions, the peak representing the ICG monomer spectral absorption maxima is at about 800 nm, however, the peak of its polymeric form is reduced to ~700 nm [17,18]. On the other hand, the concentration of the intravenously administered dye formulation quickly dilutes in a couple of seconds, so that the wavelength of the maximum absorption peak shifts toward the red-shift with a difference of 25 nm. In this framework, the broad fluorescence window of ICG emission ranges in the 750–950 nm region, and the maximum emission value at around 810–820 nm in water shifts to 830 nm in the blood [31]. Therefore, the fluorescence efficiency of ICG in blood is altered and results in just 4% of its intensity, to that of the fluorescence exhibited by fluorescein in vitro. The fluorescence yield of ICG is typically maximum at a concentration of 80 μg/mL, showing a general linear upward trend that decreases after attaining the peak at 80 μg/mL [31]. However, the emission intensity of the ICG and its lifetime can be altered by interactions with noble metal elements, such as gold [32,33,34], silver [35,36], and platinum [18]. Despite the high plasmon absorption efficiency of platinum at a wavelength of under 300 nm, the available spectral change remains unclear for the ICG long wavelength probe. In comparison with the smooth platinum surface, the emission intensity and lifetime of its rough surface reduce by approximately two-fold [18]. Referring to the study of the ICG rubber ring, LED and laser light sources make no significant difference in the ICG fluorescence [29]. However, it should be noted that the maximal absorbance of ICG slightly changes in the water (790 nm) to that of physiological fluids (e.g., 815 nm in human serum) [27]. Its stability in methanol or other organic solvents and bile (t_1/2_ > 1 year) guarantees the highest molar linear absorbance. In contrast, the rapid decomposition of ICG in duodenal fluid (t_1/2_ = 3.6 days) and distilled water (t_1/2_ = 1.4 days) generates the lowest absorbance epsilon [37].

**Figure 2:**
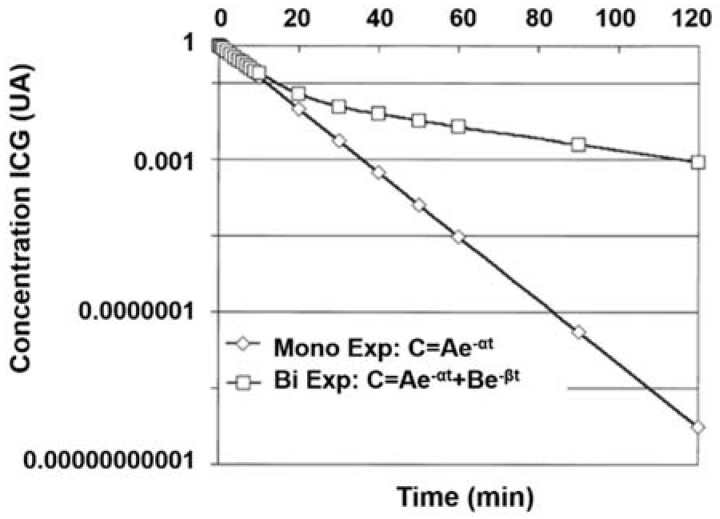
Mono- as well as bi-exponential models illustrating the decay of ICG concentration. C = Ae^−αt^ is the mono-exponential modelization, and C = Ae^−αt^ + Be^−βt^ is the bi-exponential modelization for ICG blood clearance (C: concentration, A and B: constants, α, and β: slope of clearance, t: time). Reproduced from Ref. [31], with permission from Elsevier, 2000.

In general, ICG shows distinctive metabolic features in the body. The amphipathic nature of ICG contributes to its interaction with various proteins such as lipoproteins and plasma proteins or the physical interactions with human serum albumin in blood [19]. Combined with electrophoretic methods and the fundus video imaging system, the binding properties of ICG with plasma proteins identify that it binds preferentially to the alpha-1 lipoprotein, instead of albumin [31,38]. In this framework, ICG intensely binds to the high-density lipoprotein and moderately to the low-density lipoprotein, which was confirmed by polyacrylamide gel electrophoresis [31]. The lipid portion of the plasma lipoproteins and the hydrophilic portion of the phospholipids are the most feasible sites for its binding, which cause the lack of specificity [18,31]. Once injected into the veins, the excess amount of dye is confined in the blood vessels by attaching to these proteins and lipoproteins. Moreover, the excretion of the ICG predominantly happens in its native form after being entirely delivered from the hepatic parenchymal cells into the bile ducts, leading to its swift removal [18]. The half-life in the initial phase was at around 3–4 min with the method of the mono-exponential modelization (Figure 2, Table 1) [18,31], revealing the rapid elimination from the body [22]. In contrast, the second phase adopted the bi-exponential equation for investigating the later 30 or 50 min [18,31], and was more substantial than the first. Although the removal of ICG from the plasma was very rapid, there was a distinct delay in biliary excretion [38]. In addition to the hepatic clearance effect, it was also acknowledged by Zhao et al. that the efficient uptake of the dye by pulmonary endothelial cells was also a pathway for the excretion of ICG or its associated compounds [39,40]. Furthermore, it is evident that the combination of ICG with other species (e.g., primaquine (PQ)), can avoid its lysosome-dependent metabolism, i.e., autophagy [41]—as PQ suppresses the alliance of autophagosome and lysosome, and thus the endocytosis mediated by the peptide is enhanced. These consequences demonstrate that the enhancement of selective recognition and internalization are successfully achieved.

**Figure 3:**
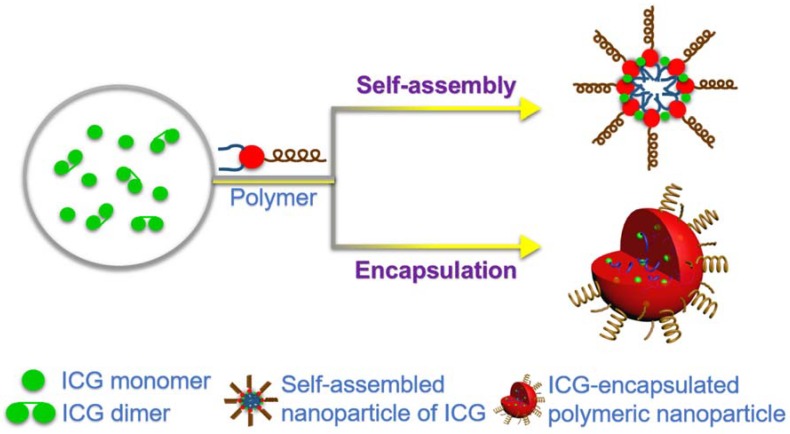
The molecular rearrangements and various delivery patterns of ICG.

Despite its significant advantages and advancements in therapeutic modalities, a molecular imaging probe in vivo shows that ICG suffers from certain limitations, such as concentration-dependent aggregation, rapid circulation clearance, poor in vitro aqueous stability, photobleaching, and photodegradation [22,53,54]. More often, the ICG dye monomers aggregate into oligomers in the aqueous environment with varying concentrations (ranging from 5 to 100 μM) [31]. Due to the increase in its concentration, transient half-life, and hydrophobicity, the maximum absorbance of ICG at 780 nm collapses, and the peak within the wavelength range of 700–720 nm indicates the resultant aggregation of the dye. However, previous reports indicated that this was not merely the result of covalent cross-linkage due to the denaturation with methyl (-CH_3_) groups [27]. Moreover, the utilization of ICG as a photosensitizer is limited due to the risk of the photo- as well as thermal- induced degradation [54]. To overcome these limitations, ICG has been encapsulated in various biodegradable polymers, which offer enormous benefits to ICG molecules such as improved stability, preventing unwanted aggregation, and a reduced self-quenching trait of its fluorescence, among others. Moreover, ICG in polymeric carriers have enormous advantages during its delivery such as improved deep-tissue penetration, lower absorption, the production of singlet oxygen in the presence of light, decomposition of the polymethine chain to yield cytotoxicity, and a minimal auto-fluorescence compared to other visible optical probes [22,55]. In addition to its encapsulation in the nano-sized polymeric architectures, ICG can also be directly injected into the bloodstream or water in the form of monomer or dimer, and self-assembled ICG nanoparticles (Figure 3). However, the applicability of its native forms is limited as they are prone to the undesired binding to lipoprotein and engulfment by the phagocytic system in the physiological fluids. On the other hand, various inorganic construct based nanocarriers have also been used to convey ICG, such as gold nanorods [32,56,57], mesoporous silica [58,59,60], and gadolinium(III)-chelated silica [61], among others [16,62,63,64,65]. On the other hand, carbon-based nanomaterials such as graphene oxide [66,67,68], liposomes [69,70,71,72,73], and folic acid [74,75], have also been applied for the delivery of ICG. Despite the success in generating these diverse systems with intrinsic functionalities for different applications, some of these materials suffer from various critical issues such as low biodegradation rates and a subsequent long-term accumulation-induced biosafety risk [76,77,78,79], which stringently limit their applicability in the biomedical field.

To this end, polymers are highly advantages in encapsulating and carrying ICG as they offer numerous advantages such as enhance its half-life, cost-effective reduce its fast degradation and increase its biocompatibility, biodegradability, fluorescence intensity, physicochemical stability, target specificity and pharmacokinetic attributes [17,27,42,54,80,81]. In addition, the incorporation of ICG in the polymeric constructs make it absorb and emit at the longer wavelength than that of the naked ICG molecules [19,82]. In this context, various polymers such as poly(d,l-lactic-co-glycolic acid) (PLGA), poly(ethylene glycol) (PEG), and poly(ε-caprolactone) (PCL) and others, have been used in encapsulating ICG for imparting the highly beneficial qualities such as selectivity, preferential localization, enhanced photoactivation, biocompatibility, and biodegradability, among others. However, it should be noted that polymer selection plays a crucial role in designing the delivery systems for delivering ICG, concerning their mutual compatibility, preparation method, and solubility in the solvent. Moreover, the polymer coating over the surface of the nanoparticulate forms of ICG, such as PEG, can make the ICG nanoparticles capable of successfully evading the uptake by a mononuclear phagocyte system and prolonging its residence time in the blood [54]. In addition, the designs could be further modified by conjugating or incorporating a specific drug in the polymeric framework, which would result in time-dependent drug release for better synergistic therapeutics [17]. In addition to polymers for its sustained delivery attribute, numerous studies have reported the liposomal formulations of ICG, where the spherical lipid membrane vesicles are encapsulated with an aqueous core containing ICG [38,55]. Various other combination strategies have also been reported to improve the delivery efficiency of ICG, such as polymeric nanoparticles containing ICG as a core and lipid shell around them. These innovative designs have provided great targeting capabilities and efficient delivery of ICG [22].

In the further sections of this review, we mainly aim to discuss the delivery of ICG that has been encapsulated in various polymers such as PLGA, PEG, poly(ethylene imine) (PEI), lipids, poly-l-lysine (PLL), PCL, and other multi-polymer systems, highlighting the critical advantages and constraints associated with them. In addition, we give insights of other drugs or antibodies involved in the nano-compounds to achieve more direct and precise biomedical duties. We then elaborate the discussion on the utilization of various polymeric nanocomposites of ICG in various biomedical applications focusing on targeted diagnosis, cancer therapeutics (PDT or PTT) [57], imaging, angiography [31,83,84,85], surgery, [39,53,86,87], microsurgical repair and reconstruction [43], Sentinel lymph node [55], ophthalmology, and cardiac or hepatic vascular systems [38]. Finally, we summarize the viewpoints and potential outlooks of these innovative nanocomposites as perspectives.

## 2. ICG-Encapsulated Polymeric Composites

As mentioned earlier, the encapsulation of ICG in the polymeric construct enhances its physicochemical attributes such as half-life, bioavailability, stability and pharmacokinetic characteristics, and reduces the ease of degradation [17,27,42,88,89]. Moreover, these versatile ICG-encapsulated NIR theranostic nanoparticles also offer numerous other advantages such as significant targeting ability and sensitivity in tumor imaging, which facilitate them in exploring other innovative therapeutic applications [44,90]. Herein, we elaborate on the discussion of ICG encapsulation and its delivery using various ground-breaking polymer nanocomposites with a set of examples.

### 2.1. PLGA

PLGA is one of the most preferred polymers for biomedical applications and has been utilized in the development of various FDA-approved therapeutic devices due to its attractive properties such as biodegradability, biocompatibility, and hydrophobicity. PLGA is generally considered for its degradation ability by hydrolysis under normal physiological conditions yielding two monomers; i.e., lactic acid and glycolic acid. These metabolic by-products play a major role in its biocompatibility. Moreover, the hydrophobic nature of this polymer impedes the ease of water exchange with the environment, which assists in the release of ICG from the polymeric framework [45,91]. Numerous efforts have been dedicated to the preparation of ICG-encapsulated PLGA composites for better therapeutics. In one case, Yang et al. synthesized ICG-entrapped PLGA that resulted in the solubility of the drug payloads and eventually protected them from early biodegradation [92,93]. On NIR ray exposure, apart from an improved photothermal effect causing hyperthermia, these composites generated enormous levels of intracellular singlet oxygen, thus leading to an enhanced PDT effect, compared with that of the unstable free ICG at an equivalent concentration. The authors claimed that these polymeric constructs significantly augmented the thermal stability and the release efficiency of ICG. Moreover, it should be noted that the combination of these substances was hypotoxic and metabolic such that it had great potential for radiation therapy with the characteristics of fluorescent stability and tumor targeting [39]. In addition to ICG, various other drug molecules; e.g., doxorubicin (DOX) [33,45,92,94,95,96], superparamagnetic iron oxide [62,63,64], hematoporphyrin [97], paclitaxel [98,99], docetaxel [100], and folate [33,48] have been encapsulated within the PLGA polymeric framework for exploring its co-delivery efficacy for biomedical target diagnosis as well as therapeutics. In an attempt to address the co-delivery efficacy of PLGA nanocomposites, Lee et al. synthesized the ICG and DOX-encapsulated PEG-b-PLGA nanocomposites in figuring out the fabrication and characterization of anti-human epidermal growth factor receptor 2 (HER2) and applied them in overcoming the drug resistance of breast carcinoma [19,70,101]. In another study, Hung et al. fabricated PLGA as a nanovehicle to specifically deliver ICG into the tumor locations along with DOX [45]. Similarly, Wang et al. filled ICG-PLGA with perfluorocarbon gas and generated microbubbles, which remarkably enhanced the NIR fluorescence signal intensity, retention time, and ultrasonographic contrast of ICG [102].

### 2.2. PEG

Based on the clinical safety studies, PEG has been acknowledged as an inert and invasive polymer in biology. More often, this effective steric stabilizer is utilized for protecting the nano-sized architectures against opsonization and phagocytosis, which are known to be the leading protective mechanisms of the body [54]. The PEGylated-ICG nanoconstructs possess numerous advantages over the non-PEGylated analogues. First, this biodegradable polymer enhances the aqueous solubility of ICG. For example, ICG is highly soluble when encapsulated in PEG (ICG-PEG-COOH) compared to that of ICG-2-mercaptophiazoline composites [22]. Second, the incorporation of ICG in PEG results in the significant enhancement of the eventual molecular weight of the construct, which would simplify the purification of the dye-polymer composites (e.g., thorough elution in Sephadex column), with the difference in solubility. In addition, the polymeric composites possessing higher Mol. Wt. would promote easy handling of ICG. In a way, the conjugates can be easily purified and recovered in good yields on the basis of protein through extensive dialysis [27]. In addition, it is expedient that PEGylated conjugates are devoid of aggregation, which could be found via ultraviolet-visible (UV-Vis) spectrophotometry and size exclusion-high performance liquid chromatography measurements. Third, hemolysis and other coagulation assays of ICG-PEG have unveiled that these polymeric constructs significantly imparted excellent hemocompatibility as well as negligible cytotoxicity and long-term storage. Fourth, ICG can overcome low vascular or tissue permeability after coating with PEG [82], indicating that it could be selectively internalized into the targeted cells [19].

**Figure 4:**
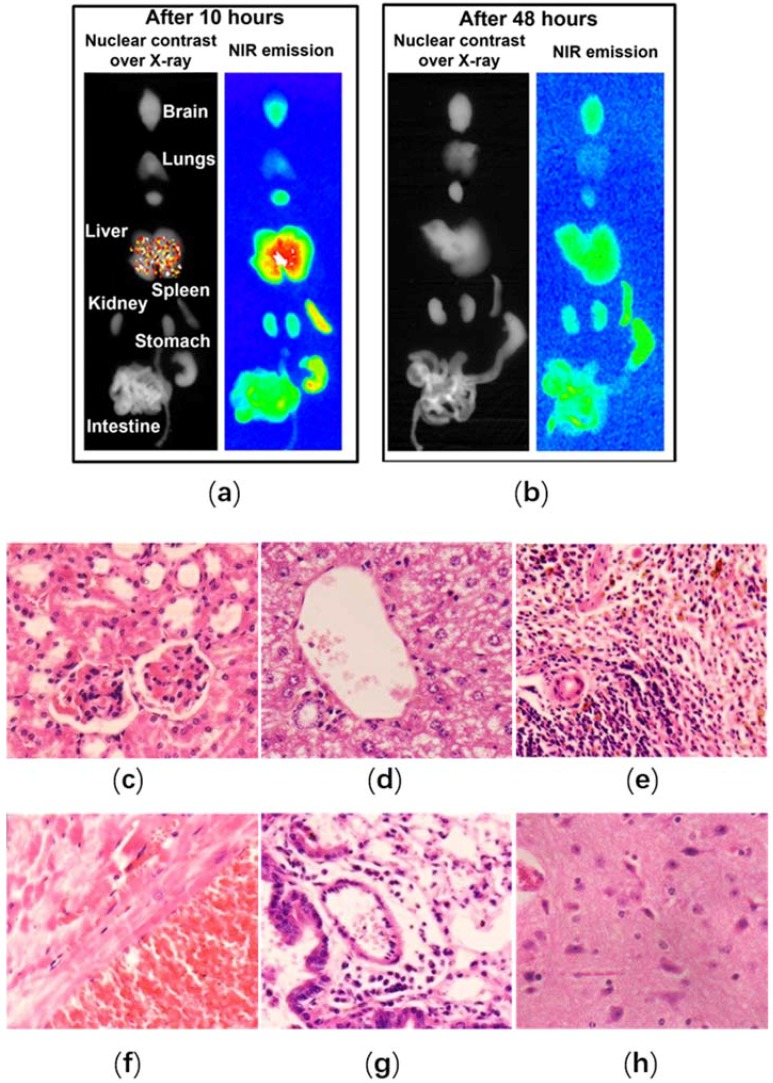
Biodistribution of ICG-loaded nanoparticles with surface modifications of polymers. Ex-vivo imaging of the major organs of mice: (**a**) 10 h after PEG-multifunctional-calcium phosphate nanoparticles (MF-nCP) injection; (**b**) 48 h after sample injection. Histological analysis of major organs as (**c**) kidney, (**d**) liver, (**e**) spleen, (**f**) heart, (**g**) lungs and (**h**) brain from mice after 48 h of sample injection at 40× magnification showing no changes in the cellular integrity or tissue morphology. Reproduced from Ref. [53], with permission from Elsevier, 2013.

In general, the nano-sized architectures of polymers significantly augment the half-life and bioavailability of ICG during the metabolism in vivo. Nevertheless, the plasma half-life of ICG is still too short due to the long blood circulation time required for targeting the tumor cells (Table 1). Activated macrophages, which can secrete inflammatory cytokines and factors generating tissue dysfunction and destruction, occupy a crucial status in the burgeoning evolution of the malignant disease [92,103]. Moreover, they have an ability of inherent phagocytosis targeted for the destination of imaging and therapy. Based on the enhanced permeation and retention (EPR) effect, the PEG-modified nanoparticles evade the phagocytosis uptake as well as their degradation in the physiological fluids, resulting in the substantial extension of retention time by offering the steric hindrance [27,42]. Upon coating with PEG, the phagocytic content of ICG-containing capsules caused by macrophages is reduced, and the time incubation is substantially extended to 360 min [104]. Preceding reports indicated that these composites were initially distributed to the major organs such as the kidney, lung, heart, liver, and spleen, and then a large amount of the dye was released. Further, the dye was excreted via the renal tubular epithelial cells and the hepatobiliary system [105]. However, the positive impact on the biodistribution in vivo (Figure 4a,b) and other pharmacokinetic properties of ICG-encapsulated PEG composites remain to be explored.

According to the preceding investigations based on Single-photon Emission Computed Tomography (SPECT) imaging, the PEGylated composites have shown an improved non-invasive tumor monitoring behavior with a relatively higher accumulation in the liver (Figure 4a) [42,53]. The scanned images of all major organs indicated that no significant influence on the tissues was observed and the normal anatomy or histology was kept consistent (Figure 4c–h) [53]. In another study, Wu et al. fabricated an innovative ICG-PEG-Ag_2_S nanoprobe system, which had presented relatively long blood retention time and the targeting feasibility of this functional nanoprobe toward the atherosclerotic lesions in mice models was verified [43]. The authors demonstrated that these PEG-modified nanoprobe systems partially got rid of the elimination from the liver, a major organ of the reticuloendothelial system, and also extended the half-life as well as the circulation time in the blood (Table 1), favoring the accumulation of nanoparticles in the targeted pathologic tissues [27,54,82]. In another study, Zhang et al. fabricated ICG-conjugated nanographene oxide (NGO) with an average size of approximately 95 nm, which had shown significant cellular internalization in human osteosarcoma cell and exhibited excellent chemotherapeutic effects to suppress the proliferation of tumor cells [17,54]. Similarly, the effective suppression of tumor growth had also been achieved by intratumoral injection in PTT of animal studies [19].

In addition to the various polymers discussed above, there has been increasing interest in preparing the hybrid nanocomposites for efficacious delivery of ICG. Along this line, phospholipids have become the common interest as they significantly augment the stability of ICG in an aqueous environment. In one case, ICG was initially immobilized over the phospholipids, and further, these conjugates were coupled with conventional polymers such as PEG; e.g., ICG-PEG-DSPE [44,106,107,108] and ICG-PEG-DOPE [109]. Moreover, ICG was also encapsulated in the PEG framework containing terminated reactive groups such as ICG-PEG-SH [110], or ICG-PEG-NH_2_ for better targeting ability [111].

### 2.3. PEI

PEI is the most commonly used cationic polymer for drug delivery due to its stability in the physiological fluids and is highly suitable for encapsulating the negatively-charged ICG [93]. While silica or PEG could not restrict the leaching-out of ICG, PEI provided extra protection to the dye and prevented its leaching-out, which could be ascribed to the electrostatic interactions between them [91]. The encapsulation of ICG in PEI offers great interest against the leakage of ICG, which was analyzed and concluded by Ashokan et al. [53]. Further, numerous studies have been dedicated to exploring the efficacy of these conjugates concerning the cellular internalization, which could reduce the nonspecific interaction and selective entanglement with the tumor-specific receptor cells; e.g., FA (for tumor cells expressing folate receptors) and ICG were conjugated to terminal and side chains of the branched poly(l-glutamic acid), respectively [59,112]. In one case, Yang et al. fabricated a core-shell based nanocomposite delivery system with superparamagnetic iron oxides and ICG in the core that were wrapped around the surface with PEI by the emulsion-diffusion-evaporation method [92]. Based on the NIR imaging, the highly sensitive, real-time tracking of the kidney in mice was explicitly recorded, including stronger ICG signals with a lower signal and contrast to noise ratio regarding the specific situation of the injured kidney in the unilateral ureteral obstruction model with obstruction of the left ureter. Moreover, these versatile nanoparticles had effectively accomplished the siRNA transfection of macrophages.

**Figure 5:**
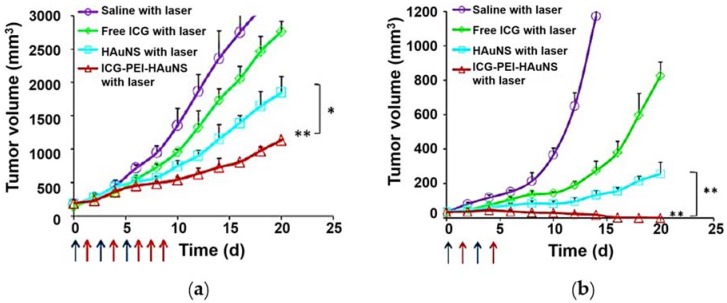
Anti-proliferative efficacy in vivo. (**a**) Tumor growth curves for mice bearing CT-26 tumors treated with saline, free ICG, HAuNS, or ICG-PEI-HAuNS, *n* = 6. The blue arrow indicates injection, and the red arrow indicates the laser exposure (1 W/cm^2^, 2 min, 120 J/cm^2^). (**b**) Inhibition of tumor metastasis. Tumor growth curves for mice bearing B16 tumors treated with saline, free ICG, HAuNS, or ICG-PEI-HAuNS, *n* = 6. The blue arrow indicates the injection, and the red arrow indicates the laser exposure (1 W/cm^2^, 2 min, 120 J/cm^2^). Reproduced from Ref. [113], with permission from American Chemical Society, 2017.

In addition to the improvement of fluorescence intensity, laser sensitivity or efficient delivery, several attempts have been made to improve the fluorescence yield of ICG upon conjugation with the polymer. In one case, Chen et al. encapsulated ICG within the polymeric micelles of thermos-sensitive Pluronic F-127 as the core, and then these constructs were cross-linked with the pH-responsive PEI [93]. The fluorescence yield of these nanocapsules was under the control of local pH change and the micelle’s size, in which the size was reversibly altered (swelling/shrinking) with a significant change in the temperature (falling/rising) [59,93]. However, this PEI cross-linked design suffered from a limitation of cytotoxicity issues resulting in the drastic cell death than that of the nanomaterial alone (Figure 5). These results demonstrated that the applicability of a cross-link was significantly limited due to the severe biocompatibility issues over other polymers such as PEG or PLGA.

In addition to the encapsulation of ICG alone, PEI has been used to coat the delivery systems that carry the ICG for therapeutics. Among various inorganic nanocontainers, silica nanoparticles have proven to be an excellent carrier for conveying therapeutic cargo due to its extensive surface area as well as pore volume to accommodate therapeutic guest molecules ranging from the small molecules to giant macromolecules like proteins, enormous functionalization surface, colloidal stability, high dispersity, unique topology, controllable pore size, tunable particle shapes and sizes, and high stability [58,114]. In this framework, ICG can be doped onto silica carriers to overcome its photobleaching and photodegradation effects, which significantly limited its applicability since conception [32,58,59,60,115]. In one case, Quan et al. encapsulated the ICG molecules ion-paired with PEI into the silica nanoparticles (diameter 50–200 nm) through the nanoparticle-assembled capsules using the Stober process [59]. This strategy had successfully reduced the self-quenching of ICG fluorescence via significantly suppressing its aggregation.

### 2.4. Lipids

**Figure 6:**
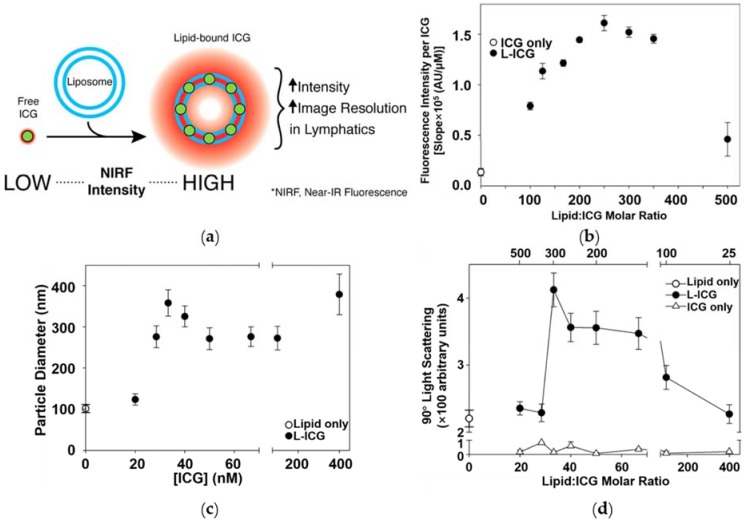
(**a**) Schematic illustration showing the preparation of lipid-bound ICG (L-ICG) and its effects; (**b**) Effects of lipid:ICG molar ratio on the fluorescence yield; (**c**) Effect of ICG concentration on the particle size analysis by photon correlation spectroscopy of liposomes; (**d**) Effect of ICG concentration on the 90° light scattering intensity of liposomes. A fixed concentration of liposomes was incubated with varying concentrations of ICG in a fixed volume. At 20 min, the mixtures were diluted to stop the reaction, and the 90° light scattering intensity was measured using a fluorometer. Reproduced from Ref. [38], with permission from American Chemical Society, 2014.

ICG encapsulation in the lipids as core-shell nanoparticles (Figure 6a) offer attractive features such as uniform dispersity, excellent stability and fluorescent intensity (Figure 6b), enhanced photothermal efficiency, targeting efficacy, metabolic distribution, optimized pharmacokinetics and insignificant toxicity with even no signs in a few instances below a certain concentration [39,40,54,116]. In their study, Kraft et al. explicitly assessed the therapeutic efficiency, light scattering (90°), fluorescence intensity and stability, light exposure, storage stability and tissue depth penetration of ICG-encapsulated liposomes [38]. The average particle diameter of the ICG-encapsulated lipids ranged from 100 to 400 nm (Figure 6c). On the other hand, the scattering intensity had shown an initial increase and reached a peak, then declined gradually with increasing ICG concentration (Figure 6d). Moreover, they observed that these lipids initially resulted in the vacant preformed liposome vesicles, triggered the aggregation to a liposome, while intermittently augmenting the light scattering intensity [38]. Preventing exposure to water directly, ICG-embedded lipids exhibited a high fluorescence intensity and long-term storage stability [17]. In addition, the equilibrium dialysis method manifested that the encapsulation efficiency of ICG was nearly complete in the liposomes (97.8 ± 0.6% of loading efficiency) [38,55]. In another study, Xin et al. prepared PLGA-lipid nanoparticles by facile self-assembly and the nano-precipitation method with covalently-conjugated ICG for tumor-targeted imaging and drug delivery in vitro as well as in vivo, which raised the coagulative necrosis and pyknosis of the tumor cells [40,117]. These innovative hybrid nanocomposites had also overcome the limitations of ICG, such as less circulation time and inefficient cellular uptake. Similarly, Zhao et al. fabricated ICG-loaded polymer-lipid nanoparticles with three different hydrodynamic dimensions by a single-step self-assembly method. These composites performed by rapidly dispersing and penetrating through the whole tumor matrix [39]. Moreover, they explored how the size of nanoparticles had affected the delivery of drugs and found that the small-sized nanoparticles were efficiently internalized by an endocytic process more rapidly in human pancreatic cancer cells [40].

In addition to the efficient delivery of ICG through cell-specific internalization in the tumor, the liposomal formulations offer numerous other benefits, such as biocompatibility enhancement, and extend the time of availability of the dye in the cells, resulting in enhanced therapeutic effects [39]. In one case, Lajunen et al. synthesized light-triggered ICG-encapsulated small-sized liposomal composites that could efficiently deliver both small as well as large drug molecules [40,116]. In contrast, with the free ICG, the exposure time to the light of liposomal ICG had extended from seconds to several hours, demonstrating the high reminisce of its fluorescence integrity. These liposomal compositions acted by prolonging the storage through minimizing the passive leakage and changes in the formulations [116]. Moreover, various modified polymers, copolymers or proteins can also be considered instead of lipids such as ICG-PEG-dextran [118], ICG-alkyne [119], ICG-cypridina luciferase [120] and ICG-avidin [121].

### 2.5. PLL

PLL has gained enormous interest for loading negatively-charged ICG molecules due to its unique structure and positive charge, which facilitate its internalization for the delivery of therapeutic cargo into the cells via prominently interacting with negatively-charged biological membranes by means of electrostatic interactions. These PLL-based composites often result in a broad absorption peak in the range of 700–850 nm, which is shorter (50 nm) than that of the resultant spectrum of the original ICG. More often, these nanocomposites are predominantly localized in the cytosol after uptake instead of penetrating into the nucleus [116]. In addition, these carriers are beneficial in loading agents for the combinatorial strategy of photothermal as well as PDT, with an excellent synergistic effect. In one case, Wang et al. fabricated polymeric nanohybrids loading ICG as well as Pt(II)-porphyrins through a modified encapsulation approach based on the precipitation method for NIR-triggered PTT as well as two-photon PDT effects. Herein, Pt(II)-porphyrins, as the photosensitizer, and organic semiconducting polymer, poly(9,9-di-*n*-octylfluorenyl-2,7-diyl) as the doping host and photonic energy donor, constituted the core of the nanoparticles inducing cancer cell death with significantly higher efficiency, while ICG-loaded PLL nanoparticles offered a strong photothermal performance [116].

In some cases, PLL can also be used as one of the co-block polymers for improving the loading efficiency of drugs, due to its surface charge attributes. Encapsulating ICG in the poly(l-lysine)-*b*-poly(l-leucine) (PLL-PLLeu) offers numerous benefits to ICG, such as significantly overcoming poor stability, improving the quantum yield, and reducing the self-aggregation of the dye [92,122]. As mentioned earlier, the PLL-based nanocomposites exert enormous intracellular localization capacity with the improvement of fluorescent stability and longer plasma half-life. Especially, the hybrid diblock copolymer with PEG shell can also regulate the cellular uptake efficiency via adjusting the length of the diblock substrates. It resulted in the impediment of ICG owing to the electrostatic adsorption and hydrophobic interactions according to Wu et al. However, they demonstrated the potential of PEG-PLL-PLLeu-ICG as a photothermal agent with excellent hyperthermia performance [122].

### 2.6. PCL

PCL is an efficient semi-crystalline polyester and an implantable biomaterial for controlled drug release due to the ease of its degradability by hydrolysis and its ester linkages in physiological conditions. Despite its significant advantages, the utility of PCL is limited, owing to stability concerns. The stability of ICG can be significantly improved by encapsulating it in a hydrophobic and biocompatible polymer. In one case, Schönbächler et al. took advantage of the properties of inorganic nanoparticles and designed an efficient approach for obtaining biocompatible silica-PCL grafted nanocomposites, which exhibited high loading amounts of ICG with excellent stability [123]. In addition, the fluorescence signal avoided the intrinsic background interference relatively, achieving the precision, accuracy, and selectivity of the detection [123,124]. Similarly, Ducray et al. prepared ICG-encapsulated silica-PCL nanocomposites for laser tissue soldering, in which the exposure of ICG-encapsulated nanoparticles had significantly affected the mitochondrial function [125]. In another study, Park et al. fabricated ICG sheath incorporated PCL fibers, which exhibited excellent NIR absorption efficiency, acceleration of the ICG release, and the substantial enhancement of the anticancer activity in conjunction with the hyperthermia effect [126]. On the other hand, ICG self-assembly into the PCL-lipid nanocomposites had significantly enriched the combinational therapy based on realizing a faster photothermal-induced release and effectively restraining the tumor growth or metastasis [127].

## 3. Biomedical Applications

**Figure 7:**
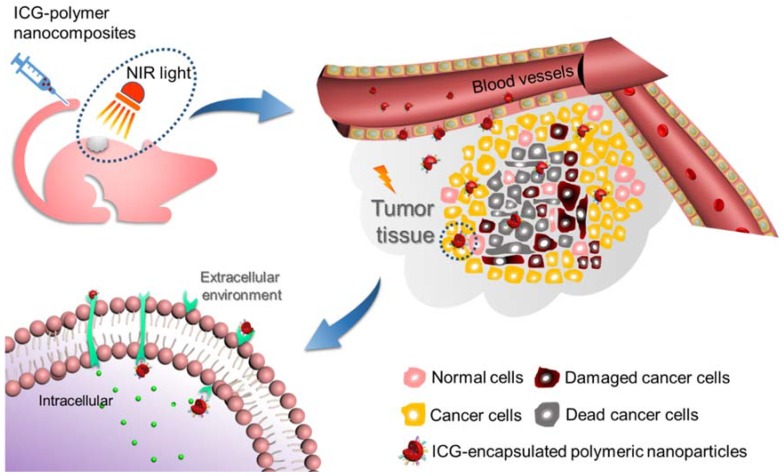
Schematic illustration showing the active photo-induced therapeutic strategy using ICG-encapsulated polymeric nanoparticles that have been used in ablating the cancer cells.

The vital cyanine dyes, herein ICG, are highly efficient fluorescent compounds that have been widely applied in various applications ranging from the spectral sensitization of photographic emulsion to nonlinear optical materials and biomedicine [128]. The applicability of ICG in the biomedical field has garnered enormous interest in the past few decades, owing to its ability to absorb light in the NIR region and efficiently perform numerous therapeutic duties. Herein, we mainly aim to discuss the biomedical utilities of various ICG-encapsulated polymeric nanoconstructs in comparison with that of the free ICG molecules with a set of examples, focusing on diagnosis, imaging, and cancer therapeutics. First, we provide a brief emphasis on their usage in the diagnosis of major diseases, like cancer, for early detection. Based on the principles of PTT and PDT, we then discuss various therapeutic strategies based on ICG-encapsulated nanocomposites for ablating the tumor cells in the presence of NIR light (Figure 7) concerning the advantages over conventional chemotherapeutic approaches or radiotherapy, which are limited by non-selectivity, high leakage, and low permeability [129,130,131,132]. With the priorities of fluorescence, safety, and synergistic effects, ICG shows enormous potential in the biomedical field over other certain materials. In an attempt to overcome multidrug resistance (MDR) in cancer, Jheng et al., fabricated the ICG bound chitosan (CS) through robust electrostatic interactions, which showed excellent photostability. Simultaneously, the dual hyperthermia effects of DOX and ICG-CS demonstrated the efficient conquering of multidrug resistance (MDR), which were usually observed in traditional therapeutics of breast cancer cells [133]. Furthermore, we are motivated to explore various other applications of ICG in biomedicine such as medical imaging with angiography, surgery, microsurgical repair, and reconstruction involving sentinel lymph node, cardiac and hepatic vascular systems due to its strong light absorption capacity at about 800 nm.

### 3.1. Diagnosis

Although ICG was developed during the Second World War, this dye has garnered enormous interest from researchers for its utilization in the field of medical diagnostics since 1956 [55,86]. ICG exhibits higher contrasted and sensitive properties in the shortwave infrared (even >1500 nm) than that of the conventional NIR range. Together with existing imaging modes, ICG shortwave infrared imaging plays a critical role in clinical applications, especially the non-invasive diagnosis of hepatobiliary clearance and lymph vessels [134]. Furthermore, the tremendous progress since its conception is evidenced by the advancements of various methods in generating ICG-conjugated constructs for their diagnostic usage. In this vein, ICG-encapsulated polymeric nanocomposites along with their conjugates with a ligand, peptide, or antibody, are the most successful strategies for accurate tumor-targeted diagnosis and treatment. Compared to free ICG solution, the accumulation of the delivered ICG from polymers into the organs was remarkably higher, demonstrating the tremendous potential of polymeric nanoparticles in tumor diagnosis and phototherapy [21,135]. For instance, the early intervention of the abnormal lymph, determination of cardiac output and blood plasma volume, evaluation of hepatic function, measurement of capillary patency and localization of tumors in tissues, and dermatology have been most successful in diagnostic applications of ICG [43,54,55,136,137]. In another case, Zhang et al. fabricated an ICG-based delivery system focusing on pharmacokinetic parameters of the released dye, which was conducive to tumor diagnosis [88].

### 3.2. Cancer Therapy

**Figure 8:**
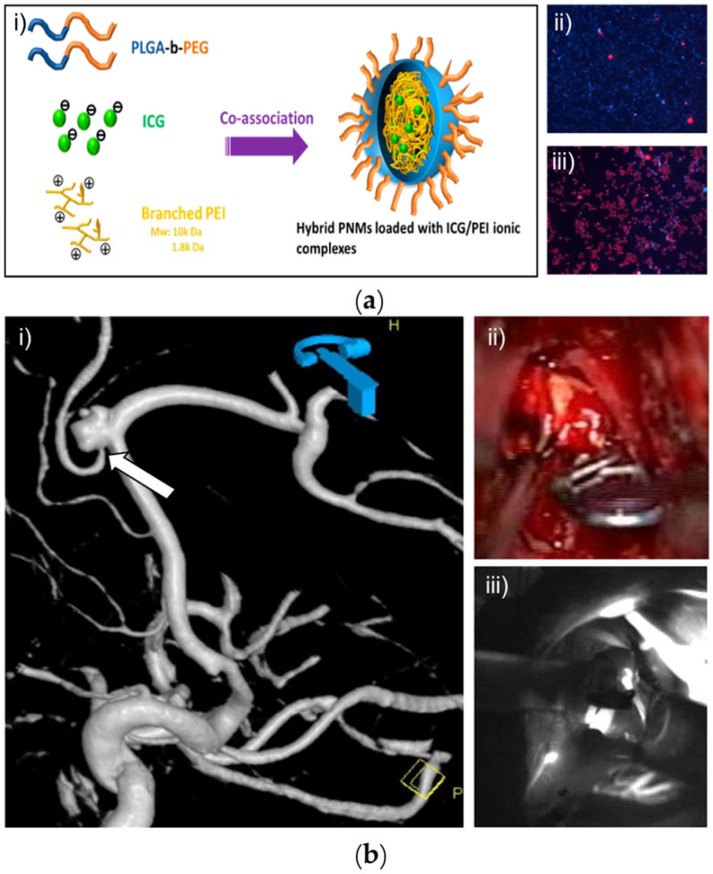
ICG-encapsulated nanoparticles for biomedical applications. (**a**) Function in PTT: (**i**) Schematic illustration showing the structure of ICG/PEI-encapsulated hybrid polymeric nanomicelles (PNMs); Fluorescence images of HeLa cells treated with free ICG (10 μM) (**ii**) or ICG/PEI-loaded PNMs (**iii**) with laser irradiation. Reproduced from Ref. [91] , with permission from American Chemical Society, 2015. (**b**) Utilization of polymeric nanocomposites in microsurgery and angiography: (**i**) Complex aneurysm of the pericallosal artery as displayed by the 3D reconstruction of DSA; (**ii**) Patency of the branching artery was analyzed by the use of intraoperative micro-Doppler after aneurysm clipping; and (**iii**) NIR ICG video angiography (ICGVA). Both methods assumed good local blood flow through a small perforating artery during surgery, which was not seen in the intraoperative cranial CT angiography (iCTA). Reproduced from Ref. [145], with permission from Springer-Verlag, 2012.

Cancer is one of the most dreadful diseases and accounts for millions of deaths globally every year due to the uncontrolled proliferation rate of tumor cells [138]. Over the past few decades, remarkable advancements have been made in comprehending the origination and development of cancer and various treatment strategies to overcome it [114,139]. In this framework, various light-induced therapeutic strategies have become more promising and as potential alternatives to the conventional treatment methods due to attractive properties such as minimal invasiveness, high selectivity, biocompatibility, and the preferential localization of photosensitizers resulting in minimal adverse effects [140,141,142]. PTT is one of the phototherapeutic strategies that predominantly harness therapeutic efficacy through hyperthermia conditions based on the synergistic therapeutic effects of efficient photosensitizer and the irradiated light at a specific wavelength (Figure 8a(i). Conceptually, under the irradiation of external light source, the excited photosensitizer at the target site transforms the optical energy at its specific absorption window of light into the precise vibrational (thermal) energy (hyperthermia) (Figure 8a(ii,iii)) [19], which ablates the targeted cancer cells. The major advantage of this approach is that it takes less time and exerts a more apparent effect but is unable to make a difference in the inner depth of tissues due to the limited transmission capacity of lasers [143]. PDT, often referred to as lucotherapy, is another light-induced therapeutic strategy, based on the light source, and a photosensitizer that targets the tissue resulting in triggered cell death through various mechanisms. One of the predominant mechanisms of PDT action is the photooxidation effect, where the light induces the generation of high amounts of deadly reactive oxygen species (ROS), which subsequently result in the ablation of intracellular organelles like nucleus and mitochondria, and eventually cell death [54,136]. PDT is generally a multi-stage process. To start with, the non-toxic photosensitizer is imposed to the tissue specimen under no light condition [27,137]. Next, when it reaches the abnormal tissue adequately, it is time to expose the photosensitizer to the laser for a certain period. Then, the photosensitive agent is activated by the sufficient energy provided by light at the targeted site, resulting in no harmful effects to the surrounding healthy tissue [54,144].

Numerous studies have demonstrated the efficacy of ICG as a potential photosensitizing agent concerning PDT as well as PTT effects. As mentioned above, ICG has shown an amplified anti-tumor effect through the photooxidation mechanism after the addition of certain oxidation agents (such as hydrogen peroxide), in human and animal cells [55,136]. In another study, Kaneko et al. examined the effect of ICG in human hepatocarcinoma cells (HuH-7 and HepG2 cell line), demonstrating that the NIR could penetrate the deeper parts of the tumor [136]. The penetration depth was reported from 10 mm to several centimeters [128,146,147]. Moreover, the major portion of the absorbed light (~88%) is converted to heat under PTT and singlet oxygen generation via PDT elicits cancer cell destruction [137]. In a study from Ma et al., ICG-encapsulated PEG-PLGA nanoparticles enhanced the delivery efficacy of ICG and achieved successful PDT treatment [27,54]. In another study, Pramanik et al. synthesized an ICG-conjugated aptamer that was immobilized onto the NGO surface for in vitro fluorescence imaging (FI), PTT, and PDT effects [148,149]. In addition, Wang et al. prepared an ICG-loaded NGO complex for PTT and PAI in vitro [4,17]. Herein, ICG-encapsulated polymers could be applied for imaging-guided PTT effect, where ICG not only represented a fluorescent marker but also acted as a light absorber [19]. Interestingly, Ren et al. developed a PEG-PCLC3-ICG nanohybrid, in which C3 ensured the efficacy of PTT and ICG was still served as a PDT agent via generating massive amounts of singlet oxygen species for a complete ablating of the oral squamous cell carcinoma [83,117].

### 3.3. Medical Imaging

In addition to the advances in photo-induced therapeutics, ICG has also been utilized for medical imaging, which is of particular interest for angiography, surgical practices, and microsurgical repair as well as reconstruction, among others. Arising from the minimum autofluorescence of deoxy- or oxy-hemoglobin, lipid, and an aqueous solution under the NIR wavelength, the fluorescence of ICG shows promising potential for biological imaging [38]. The NIR therapeutic window of ICG, with its 820 nm emission, is the only FDA-approved fluorophore for human use that leverages this property [29,38]. However, the applicability of this dye in imaging is limited due to several drawbacks, such as low sensitivity and long acquiring time of magnetic resonance imaging (MRI) and computed tomography (CT) [27,82,113]. These limitations can be easily surpassed by the ICG-incorporated polymeric nanocomposites such as ICG-PEG-Ag_2_S or ICG-PL-PEG. Moreover, they have an ability to display better performance in real-time monitoring of photoacoustic imaging (PAI) [27,43,82]. PAI is an imaging modality that typically responds to the acoustic waves generated by a pulsed laser, and provides an optical image without invasion by overcoming the limitations of ballistic depth and resolution ratio [150]. In a few instances, PAI is successfully applied during PDT for early detection and non-invasive monitoring as well as producing a much stronger signal than free ICG at its equivalent concentration [82]. Strong photoacoustic signals provide conspicuous light absorbance, clear spatial resolution, high conversion efficiency, and a reliable supervision approach under the irradiation of a NIR laser [151,152]. In one case, Zhang et al. fabricated ICG-labeled antibody molecules, which efficiently detected the cutaneous squamous cell carcinoma at deeper penetration (<1–5 cm) and high accuracy by PAI and FI as a potential probe [153]. In another study, Zanganeh et al. administered ICG-conjugated nanotubes that significantly improved the observation of an accurate tumor site and confirmed the light absorption of cancer areas at the PAI level, in the meantime, the enhanced, robust system was also confirmed by the captured fluorescent images [154]. Furthermore, Lieto et al. verified the validity of liver tumor surgery with the effective ICG-FI tool, which efficiently detected certain lesions and recognized other small tumors [155].

#### 3.3.1. Angiography

Fluorescent molecule-based angiography has been mostly used in ophthalmology for more than 40 years. ICG utilization in this approach can explicitly demonstrate the hyper- or more often hypo-fluorescence of miliary drusen by binding to its lipid component, which contributes to the observation of choroidal involvement in the inflammatory process. The decrease of ICG leakage from the choriocapillaris or the filling up with ICG to choroidal tissue can impair the impregnation of the choroidal (hypo-fluorescence) or hyper-fluorescence, and vice versa [31]. The first report to manifest the vessel visualization by ICG angiography in microsurgical subinguinal varicocelectomy was the paper that reported on the three pilot cases investigated by Shibata et al. They identified and isolated the testicular artery after intravenous injection of ICG [156]. Refining the character of ICG as a detection auxiliary to fluorescein angiography and instructing the treatment of choroidal neovascularization, it has become one of the most recent developments of ICG’s clinical application [83].

In addition to its use in ophthalmology, the ICG-based angiography approach has also been used in other fields. In one case, Ashokan et al. developed a non-toxic trimodal nano-contrast agent based on calcium phosphate nanoparticles and verified the nuclear as well as magnetic imaging and NIR imaging in vivo [53]. Unilateral revascularization procedures using ICG (Figure 8a(iii)) significantly reflected the increase/decrease of perfusion in the treated/contralateral limb and reduction in the contralateral foot circulation [84]. With the help of intraoperative ICG fluorescence angiography, Chang et al. described a method of a superior gluteal artery perforator flap that was safer, easier, and had fewer complications [43,85,147].

#### 3.3.2. Surgery

In contrast with other medical imaging modes, FI has garnered increasing interest from researchers owing to its several benefits such as higher contrast, sensitivity, resolution, and inexpensive simplicity, ensuring spatial visualization for relatively recent imaging modes [38,86]. In addition, the NIR light overcomes the poor penetration depth (100 μm) of light irradiation at the visible wavelength range. In one study, Yan et al. fabricated Arg-Gly-Asp (RGD)-modified ICG liposomes, which resulted in the high accumulation in breast tumor tissues under the observation of fluorescence signals through NIR fluorescent molecular imaging [44]. Using NIR FI and a targeting probe, a rational approach has been developed to diagnose the surgical excision [39]. Taking the Positron Emission Computed Tomography (PET)/SPECT data and intra-operational NIR images, it is feasible to conduct the image-guided surgical procedures [53].

Similarly, the microsurgical repair and reconstruction of tissues are significantly feasible with the ICG-encapsulated polymeric composites. The microsurgical process generally involves the accumulation of the conjugates in the infected areas after administration through intravenous injection. The imaging then reveals the situation where the nanoparticle is located. Eventually, the clinicians can easily determine the tumor location and take actions to repair or reconstruct the tissues (Figure 8b(i,ii)). Figure 8c illustrates that the ICG-assisted angiography might be technically applicable for all patients to display most arteries within the visual field. In this context, Nezza et al. demonstrated that the formation of dimers or multimers was highly favored to maximize ICG concentration in the aqueous solution, which was highly significant for optimizing a microsurgical intervention [43,128].

#### 3.3.3. Sentinel Lymph Node

On the basis of fluorescence and photon correlation spectroscopy, Kraft and Jeong et al. indicated that ICG had considerably interacted with the lipid membrane resulting in the ICG-encapsulated liposome nanoparticles for a higher NIR optical imaging resolution of the lymphatic system. These nanocomposite systems had offered enormous benefits, such as maximizing the fluorescence of ICG, minimizing the self-quenching, and enhanced lymphatic vessel visualization [55,157]. On the other hand, the encapsulation of ICG in liposomes exhibited excellent fluorescence intensity, which could be observed in pathogen-free mice muscle tissue beneath 0.5 cm deeper than that of the ICG monomer (1 cm), due to excellent light exposure and storage stability [38]. In contrast, the free ICG molecules could not be localized and distributed within the lymphatics after their distribution throughout the blood [38,55].

#### 3.3.4. Cardiac and Hepatic Vascular Systems

Recently, ICG has gained increasing interest in the indicator dilution technique for measuring the cardiac output [38,158]. In one case, Maarek et al. injected a small bolus of ICG to hemodialysis patients for estimating the cardiac output and blood volume, demonstrating that ICG could support the implementation of hemodynamic monitoring [159]. In another study, Martisiene et al. explored the two different ICG voltage-sensitivity mechanisms, electrochromism in the fast component and redistribution of ICG in the slow component, for monitoring the cardiac electrical behavior with the optical mapping experiment on rabbit hearts [160]. In addition, the investigations were further continued to demonstrate their suitability for evaluating the human hepatic vascular system. In an attempt to determine the hepatic extraction rates of ICG, Cherrick et al. estimated the splanchnic blood flow, using right hepatic vein catheterization in seven patients without clinical or laboratory evidence of liver disease [42]. The faster decay characteristic of ICG made a great difference during the discrimination of the degree of liver disease due to its attractive features such as higher extraction and excessive plasma retention, demonstrating that the possibility of hepatic blood flow was correlated with mild liver disease [38].

## 4. Conclusions

In summary, this review has highlighted and discussed various polymeric nanoconstructs encapsulated with ICG for various biomedical applications, focusing on therapeutics via the light-induced generation of free radicals and hyperthermia conditions, diagnosis, and medical imaging, among others. In addition, we gave a brief overview of their utilization in medical imaging, including angiography especially about ophthalmology and surgical operation. These applications have explored the ability of ICG-encapsulated polymeric nanocomposites in other biomedical applications with a better targeted diagnosis. Moreover, we also gave a brief overview of the various attractive features of ICG, including physicochemical attributes as well as metabolic features in vivo and the behavior of ICG in various streams including blood, water, and various buffered saline. These polymeric nanocomposites could efficiently overcome the drawbacks of ICG, such as short half-life, aggregation, and poor bioavailability as well as fluorescence intensity, physicochemical instability, self-quenching characteristics, poor pharmacokinetic characteristics, high degradation, and poor retention in blood.

Despite the success of various ICG-encapsulated polymeric composites including the liposome-based formulations that keep making progress in recent years, the application and their translation to clinical practice still lacks fundamental studies, such as accurate cell targeting and a more efficient targeted delivery approach. In addition to its significant efficacy in both PTT and PDT, ICG should be further explored in PAI and target treatments. Further explicit investigations are required to study the in vivo clearance of delivered ICG, in addition to the uptake by pulmonary endothelial cells and the hepatic clearance effect. We anticipate that the development of nanoparticle-based multimodal contrast agents based on ICG-encapsulated polymeric constructs with efficient targeting ability and clearance in vivo will undoubtedly find widespread applications in the fields of diagnosis and therapeutics.

## Figures and Tables

**Table 1:** Summary presenting the optical properties and biological half-life of various ICG-encapsulated polymeric nanocomposites in comparison to free ICG.

Nanoconstruct	D/nm	DC	DLC/%	DLE/%	MEW/nm	MAW/nm	Lifetime	t_1/2_	Reference
Free ICG	-	-	-	-	800	>750	3.8 ± 1.0% retained after 20 min	3.4±0.7 min	[42]
PFC-ICG	119.1 ± 25.1	6.25 μM	-	95.1 ± 2.2	825	760	>4 mth	-	[30]
HCP@PQ-ICG	81.1 ± 7.9	5 μg/mL	92.3	15.6	835	758	-	2.04 h	[41]
ICG@PEG-Ag_2_S	172.2	24 nM	-	-	1100	800	-	6.88 h	[43]
iRGD–ICG-LPs	115.91 ± 0.43	20 μg/mL	-	93.32 ± 1.25	784	801	72% left after 1 mth	-	[44]
ICG-loaded NHTPNs	78.1 ± 3.2	20 μM	6.7 ± 0.3	70.5 ± 2.4	808	796	>7 d	-	[45]
ICG@PEA112	60	10 wt.%	5.95	59.5	810	796	45% left after 1 mth	-	[46]
ICNPs	200.4	40 μg/mL	-	36.65 ± 0.02	815	780	1440 min	7–14 times than free ICG	[47]
FA-ICG-PLGA-lipid NPs	102.4 ± 4	2.5 μg/mL	-	-	801	-	84.1% left after 1 mth	-	[48]
HSA-ICG NPs	75 ± 2.4	0.2 mg/mL	-	-	808	785	>7 d	2.86 h	[49]
CL16	122.77 ± 4.07	-	1.6	-	820	785	-	5.8 h	[50]
ICG@UA/PTX NPs	130.8 ± 0.20	200 μg/mL	-	96.88 ± 2.6	800	804	>20 d	-	[51]
PIN	246 ± 11	20–75 μg/mL	-	48.75 ± 5.48	810	780	0.30 ± 0.01 ns	-	[52]

**Abbreviations**: CL16—Cisplatin-loaded liposomal ICG; D—Diameter; DC—Dye concentration; DLC—Drug loading content; DLE—Drug loading efficiency; FA-ICG-PLGA-lipid NPs—FA receptor targeted, ICG dye-doped poly(d,l-lactide-co-glycolide) lipid nanoparticles; HCP@PQ-ICG—Primaquine and ICG co-loaded cascade-targeting nanocapsule; HSA-ICG NPs—human serum albumin-ICG nanoparticles; ICG@UA/PTX NPs—ICG@ursolic acid/paclitaxel nanoparticles; ICNPs—ICG-loaded and cancer cell membrane-coated nanoparticles; iRGD–ICG-LPs—Internalized CRGDKGPDC amino acid sequence-modified ICG liposomes; MAW—Maximum absorbance wavelength; MEW—Maximum emission wavelength; NHTPNS—*N*-Acetyl histidine modified d-α-tocopheryl polyethylene glycol 1000 succinate; PFC-ICG—Perfluorocarbon-ICG nanoemulsions; PIN—PLGA-ICG nanoparticles; t_1/2_—Circulation half-life in blood.
